# Validity of Footprint Analysis to Determine Flatfoot Using Clinical Diagnosis as the Gold Standard in a Random Sample Aged 40 Years and Older

**DOI:** 10.2188/jea.JE20140082

**Published:** 2015-02-05

**Authors:** Salvador Pita-Fernández, Cristina González-Martín, Teresa Seoane-Pillado, Beatriz López-Calviño, Sonia Pértega-Díaz, Vicente Gil-Guillén

**Affiliations:** 1 Clinical Epidemiology and Biostatistics Research Group, Instituto de Investigación Biomédica de A Coruña (INIBIC), Complexo Hospitalario Universitario de A Coruña (CHUAC), SERGAS, Universidade da Coruña, A Coruña, Spain; 2 Clinical Epidemiology Research Group, Health Sciences Department, Escuela Universitaria de Enfermería y Podología, Universidade da Coruña (UDC), Campus de Ferrol, Ferrol, Spain; 3 Department of Clinical Medicine, Miguel Hernandez University, Alicante, Spain

**Keywords:** flatfoot, podiatry, validation studies, diagnostic techniques and procedures, adults

## Abstract

**Background:**

Research is needed to determine the prevalence and variables associated with the diagnosis of flatfoot, and to evaluate the validity of three footprint analysis methods for diagnosing flatfoot, using clinical diagnosis as a benchmark.

**Methods:**

We conducted a cross-sectional study of a population-based random sample ≥40 years old (*n* = 1002) in A Coruña, Spain. Anthropometric variables, Charlson’s comorbidity score, and podiatric examination (including measurement of Clarke’s angle, the Chippaux-Smirak index, and the Staheli index) were used for comparison with a clinical diagnosis method using a podoscope. Multivariate regression was performed. Informed patient consent and ethical review approval were obtained.

**Results:**

Prevalence of flatfoot in the left and right footprint, measured using the podoscope, was 19.0% and 18.9%, respectively. Variables independently associated with flatfoot diagnosis were age (OR 1.07), female gender (OR 3.55) and BMI (OR 1.39). The area under the receiver operating characteristic curve (AUC) showed that Clarke’s angle is highly accurate in predicting flatfoot (AUC 0.94), followed by the Chippaux-Smirak (AUC 0.83) and Staheli (AUC 0.80) indices. Sensitivity values were 89.8% for Clarke’s angle, 94.2% for the Chippaux-Smirak index, and 81.8% for the Staheli index, with respective positive likelihood ratios or 9.7, 2.1, and 2.0.

**Conclusions:**

Age, gender, and BMI were associated with a flatfoot diagnosis. The indices studied are suitable for diagnosing flatfoot in adults, especially Clarke’s angle, which is highly accurate for flatfoot diagnosis in this population.

## INTRODUCTION

Flatfoot is a complex foot deformity that is commonly seen in clinical practice. The flatfoot deformity is characterized by a combination of a collapse of the medial longitudinal arch, foot abduction at the talonavicular joint, and hindfoot valgus (subtalar joint eversion).^1^^,^^2^

Different procedures can be used to diagnose flatfoot, such as clinical diagnosis,^3^ X-ray studies,^4^ and footprint analysis.^5^ Footprint analysis by a pedograph is a simple, quick, cost-effective, and readily available method. Three measurements are normally used for footprint diagnosis: Clarke’s angle,^6^ the Chippaux-Smirak index,^7^ and the Staheli index.^8^ The fundamental premise of these indices is that the height of the arch is related to the footprint. In contrast, clinical diagnoses using a podoscope require the intervention of experienced clinicians.^1^

Variability in clinical practice affects podology. Flatfoot is regularly diagnosed using a podoscope. However, there are other indices that can be used to make the diagnosis. Various articles have been published on the concordance between these indices,^9^^,^^10^ and the validity of the different indices has even been determined using diagnosis carried out with a podoscope on children as a reference group^11^; however, to the best of our knowledge there are no publications that address the validity of these indices in an adult population.

The aim of the present study was to determine the prevalence and variables associated with the clinical diagnosis of flatfoot and to evaluate the diagnostic accuracy of these indices (Clarke’s angle, Chippaux-Smirak index, and the Staheli index) for diagnosing flatfoot in a clinical setting, using the clinical diagnosis with a podoscope as a gold standard, in a random sample of patients aged 40 and older.

## METHODS

### Setting and study population

A cross-sectional study was conducted between November 2009 and July 2012 on a random sample from the district of Cambre, A Coruña, Spain.

### Sampling, recruitment and inclusion criteria

The sampling population consisted of individual residents in Cambre identified through the National Health System card census. In Spain, the National Health System has universal coverage, and almost all Spanish citizens are beneficiaries of public healthcare services. The inclusion criteria were being 40 years of age or older and having provided informed consent. The sample was randomly selected after stratification by age and gender. The participants were sent a personal letter explaining the purpose of the study and the examinations that would be carried out. They were then contacted by telephone in order to arrange an appointment at the health centre.

The response rate for the group of participants aged 40–64 was 74.8%, and in the ≥65-year age group was 65.0%. Around 33% of non-responses were due to the impossibility of finding people using the healthcare identification information provided by the National Health System. The majority of non-responders in the oldest age group had died prior to the appointment.

### Sample size calculation

The sample size was calculated taking into account the total population of the municipality (*n* = 23 649). After stratification by age and gender, 1002 persons were included in the study. This sample size (*n* = 1002 persons; 505 aged 40–64 years, and 497 aged ≥65 years) makes it possible to estimate the parameters of interest with a confidence of 95% (α = 0.05) and a precision of ±5%, assuming an information loss of 15%.

### Measurements

For each person included in the study, the following variables were examined: anthropometric variables (age, gender, and body mass index), chronic comorbid diseases (using the Charlson’s comorbidity index^12^), quality of life (using the SF-36 questionnaire adapted and validated for Spain by Alonso et al^13^), dependency and independency in basic and instrumental activities of daily living (using the Lawton and Barthel indices^14^^,^^15^), and findings from a podiatric examination. The podiatric examination, performed by an experienced podologist, included study of the footprint using a pedograph and a podoscope.

**Figure 1.**
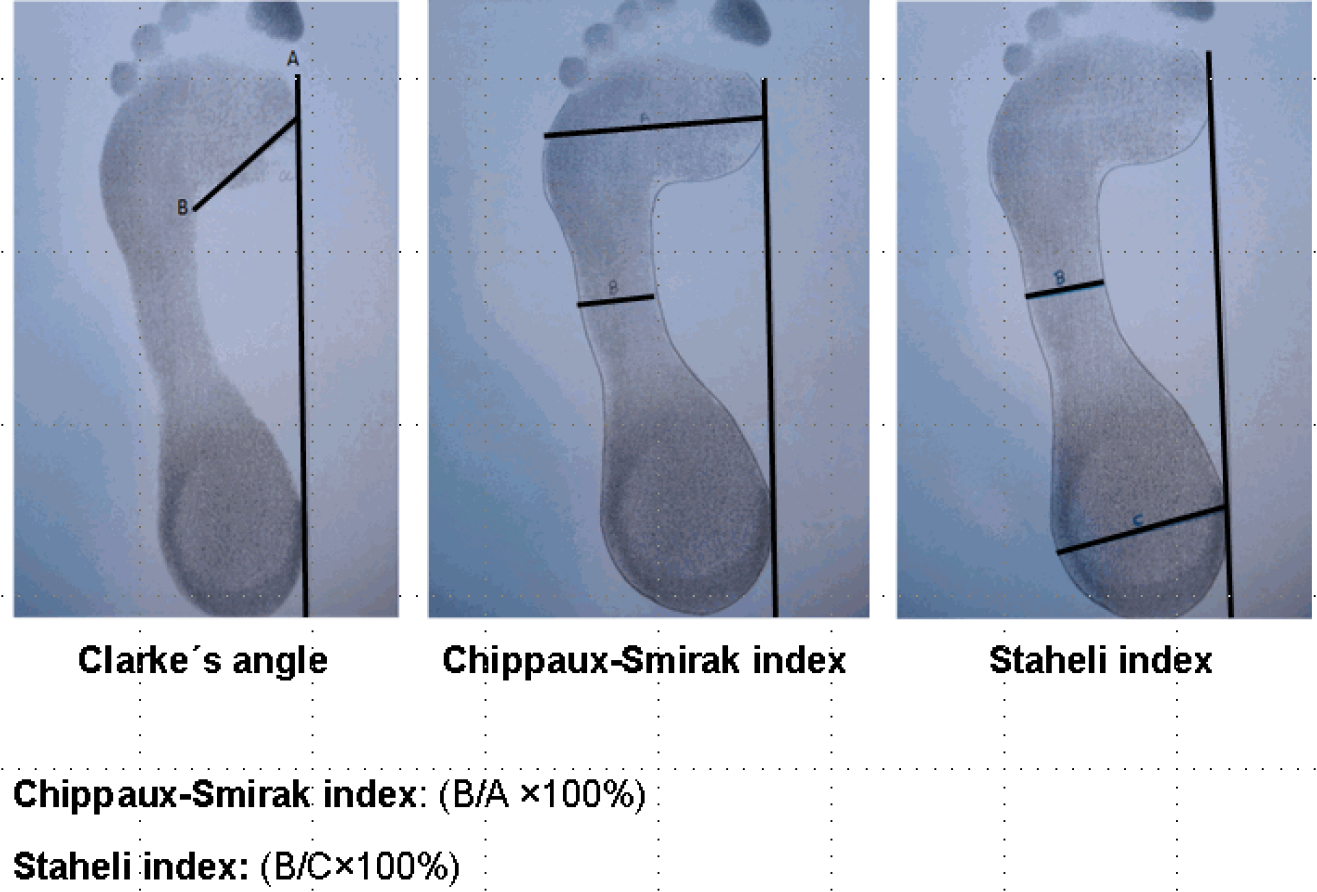
Measurement of Clarke’s angle, the Chippaux-Smirak index, and the Staheli index

The pedograph footprints were obtained by placing a reticulated piece of rubber sheeting, tensed and impregnated with ink, between the subject’s foot and a piece of stretched paper. In order to obtain the footprint, a footprint ink mat was used (podograph). To study the footprint by pedograph, three footprint measurements were used: Clarke’s angle, the Chippaux-Smirak index, and the Staheli arch index^6^^,^^8^ (Figure 1). Clarke’s angle is obtained by calculating the angle of a first medial tangential line that connects the medial edges of the first metatarsal head and the heel, and the second line that connects the first metatarsal head and the acme of the medial longitudinal arch concavity.^11^ The Chippaux-Smirak index is defined as the ratio of the length of line B, a line parallel to A at the narrowest point on the foot arch, to the length of line A, the maximum width at the metatarsals (B/A × 100, %).^11^ Finally, the Staheli arch index is the ratio of the length of line B to the length of line C, the maximum width of the heel area (B/C × 100, %).^11^

The podoscope is a simple diagnostic device that makes it possible to study the footprint. The methacrylate podoscope is a device that incorporates a fluorescent light.^4^ The clinical diagnosis of flatfoot was determined according to the weight-bearing position, when the medial arch was not visible or the medial border of the foot was convex.^3^

### Statistical analysis

The quantitative variables were expressed as mean (standard deviation [SD]), while the qualitative variables were expressed as an absolute value (*n*) and the percentage, with estimation of the 95% confidence interval (CI). Comparisons for quantitative variables were made using Student’s *t*-test or the Mann-Whitney U test, depending on which was appropriate after checking for normality using the Kolmogorov-Smirnov test. Associations between qualitative variables were analysed using Pearson’s chi-squared test. A logistic generalized mixed model was used to identify variables independently associated with flatfoot, since the present data include two measurements per subject (left and right foot). In order to study the validity of the most commonly used footprint analysis methods for diagnosing flatfoot, the left and right feet of each person were treated independently, and a total of 2004 footprints of persons aged ≥40 years were obtained.

The results of Clarke’s angle and the Chippaux-Smirak and Staheli indices were compared with clinical diagnosis of flat feet as a gold standard and displayed on a receiver operating characteristic (ROC) curve. We have used the same methodology as described by Chen et al for the statistical analysis.^11^ For each method, the ROC area under the curve (AUC) was computed, and its optimum cut-off point was determined by the Youden index. The Youden index (J) is defined as the maximum vertical distance between the ROC curve and the diagonal or change line and calculated as J = max (sensitivity + specificity − 1).^16^^,^^17^ Using the cut-off points determined by assessing the ROC curves, the positive and negative predictive values of the diagnostic tests were calculated. In order to determine the diagnostic accuracy of the tests, their likelihood ratios were calculated.^18^^,^^19^

### Ethics

The study complies with the principles laid down in the Declaration of Helsinki. Informed consent was obtained from all the participants in the study. Confidentiality was preserved in accordance with the current Spanish Data Protection Law (15/1999). The study received written approval from the regional Ethics Committee for Clinical Research (code 2008/264 CEIC Galicia).

## RESULTS

The prevalence of flatfoot in the left footprint using the podoscope was 19.0%, and that in the right foot was 18.9%, with prevalence in both feet increasing with age. The characteristics of the patients, stratified by whether they were found to be flatfoot or not after exploration with the podoscope, are shown in Table 1. The bivariate analysis revealed that patients with flatfoot, in comparison with those without, tended to be significantly older (67.3 [12.6] years versus 61.0 [12.9] years), have higher mean comorbidity according to the Charlson’s score (2.8 [1.7] versus 2.0 [1.7]), have a higher BMI (31.5 [5.2] kg/m^2^ vs. 28.5 [4.3] kg/m^2^), and have a higher waist-to-hip ratio (1.0 [0.1] vs. 0.9 [0.1]). In turn, we found that the mean foot width was discretely and significantly higher in persons with flatfoot in comparison to those without (9.6 [0.6] cm vs. 9.4 [0.7] cm). The prevalence of flatfoot increased progressively as BMI increased. Prevalence in the population with normal weight was 9.2%, increasing to 16.7% in people who were overweight and 31.4% in those who were obese. There were no significant differences in terms of the prevalence of flatfoot with respect to gender, although women had higher prevalence of flatfoot than men (22.8% vs. 19.2%).

**Table 1.** Characteristics of the study sample

	Total (*n* = 1002)	Clinical diagnosis of flatfoot		*P*

		Yes	No	
		
	Mean (SD)	Mean (SD)	Mean (SD)	
Age (years)	62.3 (13.1)	67.3 (12.6)	61.0 (12.9)	<0.001
Charlson’s comorbidity index	2.2 (1.8)	2.8 (1.7)	2.0 (1.7)	<0.001
BMI (kg/m^2^)	29.2 (4.7)	31.5 (5.2)	28.5 (4.3)	<0.001
Perimeter waist (cm)	96.4 (12.7)	101.9 (13.1)	94.9 (12.1)	<0.001
Perimeter hip (cm)	102.4 (8.5)	104.7 (9.2)	101.7 (8.1)	<0.001
Waist-to-hip ratio	0.9 (0.1)	1.0 (0.1)	0.9 (0.1)	<0.001
Forefoot width (cm)	9.5 (0.7)	9.6 (0.6)	9.4 (0.7)	0.02

	*n* (%)	*n* (%)	*n* (%)	

BMI Categories				<0.001
Normal weight (18.5 kg/m^2^ ≤ BMI < 25 kg/m^2^)	187 (18.8)	17 (9.2)	167 (90.8)	
Overweight (25 kg/m^2^ ≤ BMI < 30 kg/m^2^)	416 (41.8)	69 (16.7)	344 (83.3)	
Obese (BMI ≥ 30 kg/m^2^)	393 (39.5)	122 (31.4)	266 (68.6)	
Gender				0.16
Male	471 (47.0)	89 (19.2)	375 (80.8)	
Female	531 (53.0)	120 (22.8)	406 (77.2)	

SF-36	Mean (SD)	Mean (SD)	Mean (SD)	*P*

Physical summary index	52.5 (9.1)	51.6 (9.7)	52.8 (8.9)	0.12
Mental summary index	50.8 (8.8)	51.0 (8.8)	50.7 (8.7)	0.70
Barthel index	97.2 (6.7)	97.5 (4.4)	97.2 (6.9)	0.56
Lawton index	6.2 (1.7)	6.4 (1.7)	6.2 (1.7)	0.29

BMI, body mass index; SD, standard deviation.

Taking into account all of the variables that were significant in the bivariate analysis, we created a logistic regression model in which we found that the variables that were independently associated with a flatfoot diagnosis were age (OR 1.07; 95% CI, 1.01–1.14), female gender (OR 3.54; 95% CI, 1.26–10.01), and BMI (OR 1.39; 95% CI, 1.26–1.53) (Table 2). The presence of flatfoot was not associated with quality of life or the dependence on others for basic and instrumental activities of daily living.

**Table 2.** Mixed-effects logistic regression to identify factors associated with flatfoot adjusting for different variables

Variables	B	E.E	*P*	OR	95% CI
Age (years)	0.071	0.031	**0.024**	1.073	(1.009; 1.141)
Gender (female)	1.267	0.529	**0.017**	3.549	(1.258; 10.011)
BMI (kg/m^2^)	0.329	0.050	**<0.001**	1.390	(1.261; 1.532)
Waist-to-hip ratio	0.827	2.550	0.746	2.286	(0.015; 338.883)
Charlson score	0.080	0.226	0.724	1.083	(0.696; 1.586)
Forefoot width (cm)	0.539	0.344	0.117	1.714	(0.874; 3.362)
Foot (left)	−6.13e^−19^	0.221	1.000	1	(0.648; 1.542)
Constant	−25.754	4.235	<0.001	—	—

Random-effects parameters	Estimate	E.E	*P*		
Patients			**<0.001**		
var(constant)	16.271 35	2.204 196			(12.477; 21.219)

B, regression coefficient; BMI, body mass index; CI, confidence interval; OR, odds ratio; SE, standard error.

**Figure 2.**
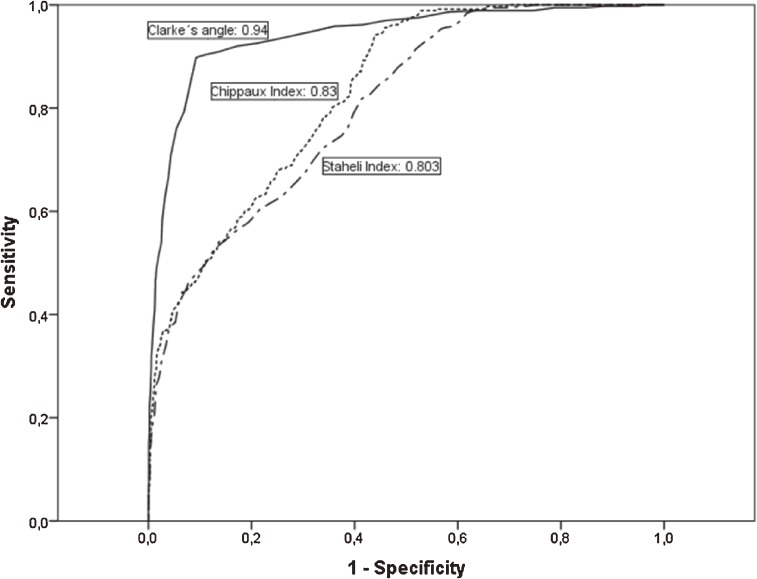
Receiver operating characteristic curve for three kinds of footprint analyses to identify factors associated with flatfoot

The ROC curves for the methods of footprint analysis are displayed in Figure 2. The area under the curve shows that Clarke’s angle had high accuracy for predicting flatfoot (AUC 0.94), followed by the Chippaux-Smirak index (AUC 0.83) and the Staheli index (AUC 0.80), which were moderately accurate. The AUC of the Clarke’s angle was significantly different from that of the Chippaux-Smirak index and the Staheli index (Table 3). The Youden index, corresponding cut-off points, sensitivity, specificity, positive and negative predictive values, and positive and negative likelihood ratios, stratified by age group, are shown in Table 3. From the ROC curve, the optimal cut-off points of these tests for diagnosing flatfoot in the total sample were determined as follows: Clarke’s angle ≤30.5°, Chippaux-Smirak index ≥45.75%, Staheli index ≥0.825%, with sensitivities of 89.8%, 94.2%, and 81.8% respectively. The positive predictive values for Clarke’s angle, the Chippaux-Smirak index, and Staheli index were 69.5%, 33.5%, and 31.7% respectively. Respective negative predictive values were 97.4%, 97.6%, and 93.2%. Clarke’s angle was found to have the highest positive likelihood ratio (10.54).

**Table 3.** Cut-off points and statistical parameters for three footprint analysis methods to identify factors associated with flatfoot, using clinical diagnosis as a gold standard

*40–64 years*	Clarke’s angle	95% CI	Chippaux-Smirak index	95% CI	Staheli index	95% CI
Youden Index	0.758	(0.69–0.83)	0.462	(0.39–0.52)	0.372	(0.30–0.44)
Cut-off point	31.50		45.05		0.98	
AUC	0.928	(0.899–0.957)	0.802	(0.763–0.842)	0.778	(0.746–0.810)
Sensitivity	83.76%	(75.54–89.70)	87.18%	(79.43–92.41)	54.07%	(47.62–60.38)
Specificity	92.05%	(89.96–93.75)	58.36%	(54.94–61.70)	83.19%	(80.23–85.88)
PPV	59.39%	(51.46–66.88)	22.52%	(18.81–26.70)	52.99%	(46.62–59.27)
NPV	97.61%	(96.22–98.51)	97.04%	(95.06–98.27)	83.83%	(80.85–86.44)
Prevalence	12.19%	(10.22–14.46)	12.19%	(10.22–14.46)	25.95%	(23.16–28.83)
PLR	10.54	(8.26–13.44)	2.09	(1.88–2.33)	3.23	(2.64–3.94)
NLR	0.18	(0.12–0.27)	0.22	(0.14–0.35)	0.55	(0.48–0.63)

*≥65 years*	Clarke’s angle	95% CI	Chippaux-Smirak index	95% CI	Staheli index	95% CI

Youden Index	0.713	(0.66–0.76)	0.420	(0.35–0.45)	0.373	(0.30–0.44)
Cut-off point	30.50		46.03		0.98	
AUC	0.910	(0.888–0.931)	0.788	(0.757–0.819)	0.778	(0.746–0.810)
Sensitivity	86.59%	(81.53–90.46)	89.84%	(85.20–93.19)	54.07%	(47.62–60.38)
Specificity	84.76%	(81.83–87.29)	50.14%	(46.38–53.90)	83.19%	(80.23–85.88)
PPV	66.56%	(61.06–71.66)	38.70%	(34.72–42.85)	52.99%	(46.62–59.27)
NPV	94.75%	(92.62–96.30)	93.37%	(90.24–95.58)	83.83%	(80.85–86.44)
Prevalence	25.95%	(23.21–28.89)	25.95%	(23.21–28.89)	25.95%	(23.16–28.83)
PLR	5.68	(4.74–6.81)	1.80	(1.65–1.96)	3.23	(2.64–3.94)
NLR	0.16	(0.12–0.22)	0.20	(0.14–0.30)	0.55	(0.48–0.63)

AUC, area under the receiver operating characteristic curve; CI, confidence interval; NLR, negative likelihood ratio; NPV, negative predictive value; PLR, positive likelihood ratio; PPV, positive predictive value.

## DISCUSSION

The present study found that the prevalence of flatfoot as assessed using a podoscope was 19.0% in the left footprint (21.5% in women and 16.2% in men) and 18.9% in the right footprint (19.8% in women and 17.9% in men). These findings are consistent with those of other studies in relation to the prevalence of flatfoot, which reported prevalence of 19.0% (20.1% in women and 17.2% in men) in one study^20^ and 20% in women and 17% in men in another study.^21^

BMI, female gender, and age were associated with the prevalence of flatfoot in our study. Some studies describe how podological pathologies increase with age,^22^ while others describe how flatfoot decreases with age after adjusting for other covariables^23^ or indicate that neither age, gender, nor BMI are related to flatfoot.^24^ One study carried out in elementary schools identified gender and being overweight as risk factors for flatfoot,^25^ while studies among adolescents^26^ and children of pre-school age^27^ identified flatfoot as being associated with increased BMI.

Similar findings have been published for studies with adult populations. One study in an adult population with self-reported flatfoot deformity showed associations between flatfoot and age, male gender, and BMI.^23^ One study reported an association between flatfoot and obesity,^28^ while another found that obesity is not associated with flatfoot.^25^ The higher prevalence of flatfoot in women identified in our study is consistent with other randomized studies, such as the study described by Dunn.^20^

We also found that the presence or absence of flatfoot does not modify quality of life. Quality of life has been used as a measurement of results for surgical treatment of the feet. Some authors have not found any differences in quality of life based on the pathology detected.^29^ However, a 6-year follow-up of the North Staffordshire Osteoarthritis Project found a progressive reduction in all SF-36 component scores as the severity of hallux valgus increased.^30^ The lack of significant findings on the SF-36 may be due to the generic nature of the outcome measured, although the SF-36 has been described as a relevant tool for capturing changes in outcome after hallux valgus surgery.^31^ Quality of life studies in individuals with chronic foot conditions have generally focused on patient satisfaction following surgery and have not assessed the impact of foot conditions themselves on quality of life.

Likewise, flatfoot was not associated with deterioration of instrumental activities of daily living. Consistent with our results, other authors have indicated that general physical functioning and participation in physical activity were not adversely affected by the presence of podiatric pathology.^32^ Similar results were recently described, without significant differences between foot conditions (hallux valgus, hallux rigidus and hammer toe), using age- and gender-matched controls.^29^

One of the aims of the present research was to study the validity of the most commonly used footprint analysis methods for diagnosing flatfoot, using clinical diagnosis as a gold standard. The Staheli index, Clarke’s angle, and the Chippaux-Smirak index are regarded as reliable by many researchers.^33^^,^^34^ Our study shows that the indices we studied are suitable for the diagnosis of flatfoot in the adult population, and that all of them have high sensitivity. Of the three methods we assessed, Clarke’s angle had a higher specificity (90.7%) than the Chippaux-Smirak index (56.1%) and the Staheli index (58.7%). Some authors have described the following as the cut-off points for the diagnosis of flatfoot: Clarke’s angle ≤30°; Chippaux-Smirak index >45%; Staheli index >0.69%.^35^^,^^36^ Our findings suggest that the cut-off points determined by the Youden index for the diagnosis of flatfoot should be ≤30.5° for Clarke’s angle, >45.8% for the Chippaux-Smirak index, and >0.83% for the Staheli index.

The area under the ROC curve for the diagnosis of flatfoot shows that the highest value was for Clarke’s angle (0.93). Other authors^11^ have shown that the Chippaux-Smirak index (0.95) is highly accurate in diagnosing flatfoot in preschool children. Our analysis showed that the overall performance of Clarke’s angle was superior to that of the other tests in a random sample aged ≥40 years.

In our study, a flatfooted person is 9.7 times more likely to have a Clarke’s angle ≤30.5° than a non-flatfooted person. A negative likelihood ratio of 0.11 indicates that a flatfooted person is 0.11 times less likely to have a negative test result than a non-flatfooted person. These findings demonstrate that Clarke’s angle is highly accurate in the diagnosis of flatfoot in our sample.

The prevalence of flatfoot in our study is consistent with randomized studies carried out in other areas, and older age, female gender, and higher BMI are associated with flatfoot. We also found that footprint analysis methods are suitable for diagnosing flatfoot, with Clarke’s angle being the most accurate.
